# Mycotoxin-Caused Intestinal Toxicity: Underlying Molecular Mechanisms and Further Directions

**DOI:** 10.3390/toxics13080625

**Published:** 2025-07-26

**Authors:** Tian Li, Weidong Qiao, Jiehong Zhou, Zhihui Hao, Gea Oliveri Conti, Tony Velkov, Shusheng Tang, Jianzhong Shen, Chongshan Dai

**Affiliations:** 1 State Key Laboratory of Veterinary Public Health and Safety, Key Laboratory for Detection of Veterinary Drug Residues and Illegal Additives of Ministry of Agriculture and Rural Affairs, College of Veterinary Medicine, China Agricultural University, Beijing 100193, China; 2 Technology Innovation Center for Food Safety Surveillance and Detection (Hainan), Sanya Institute of China Agricultural University, Sanya 572025, China; 3 Department of Medical, Surgical Sciences and Advanced Technologies “G.F. Ingrassia”, University of Catania, Catania 95123, Italy; 4 Department of Pharmacology, Biodiscovery Institute, Monash University, Clayton, VIC 3800, Australia

**Keywords:** mycotoxin, intestinal health, toxicity mechanisms, micro-RNA

## Abstract

Mycotoxins represent a group of highly toxic secondary metabolites produced by diverse fungal pathogens. Mycotoxin contaminations frequently occur in foods and feed and pose significant risks to human and animal health due to their carcinogenic, mutagenic, and immunosuppressive properties. Notably, deoxynivalenol, zearalenone, fumonisins (mainly including fumonisins B1, B2, and FB3), aflatoxin B1 (AFB1), and T-2/HT-2 toxins are the major mycotoxin contaminants in foods and feed. Undoubtedly, exposure to these mycotoxins can disrupt gut health, particularly damaging the intestinal epithelium in humans and animals. In this review, we summarized the detrimental effects caused by these mycotoxins on the intestinal health of humans and animals. The fundamental molecular mechanisms, which cover the induction of inflammatory reaction and immune dysfunction, the breakdown of the intestinal barrier, the triggering of oxidative stress, and the intestinal microbiota imbalance, were explored. These signaling pathways, such as MAPK, Akt/mTOR, TNF, TGF-β, Wnt/β-catenin, PKA, NF-kB, NLRP3, AHR, TLR2, TLR4, IRE1/XBP1, Nrf2, and MLCK pathways, are implicated. The abnormal expression of micro-RNA also plays a critical role. Finally, we anticipate that this review can offer new perspectives and theoretical foundations for controlling intestinal health issues caused by mycotoxin contamination and promote the development of prevention and control products.

## 1. Introduction

Filamentous fungi are extensively spread throughout nature and they have a high species diversity and exhibit strong environmental adaptability [1]. Secondary metabolites produced by filamentous fungi, commonly known as mycotoxins, have been extensively documented. To date, over 400 mycotoxins have been identified. Among these, aflatoxins (AFs), ochratoxins (OTA), type-A trichothecenes (such as T-2, HT-2, neosolaniol [NEO], and trichodermin, 4,15-diacetoxyscirpenol [DAS]), type-B trichothecenes (including nivalenol [NIV], and trichothecin [TCN], and deoxynivalenol [DON]), and zearalenone (ZEN), and fumonisins (FUMs), citrinin, ergot alkaloids, and patulin stand out as the most prominent compounds associated with various health disorders in humans and animals [2,3]. Currently, mycotoxin-contaminated animal feed is frequently detected across the world. A global survey of mycotoxin contamination in 4601 samples in 2023 showed that DON is the most frequently found in all samples and finished feed, with the corresponding detection rates being as high as 70% and 75%, respectively, among the emerging regulated or guideline mycotoxins; the top five in all samples from various countries are DON, ZEN, FUMs (including fumonisin B1 [FB1], FB2, and FB3), aflatoxin B1 (AFB1), and T-2/HT-2 toxin [4]. In detail, DON, known as vomitoxin, is a harmful secondary metabolite produced by *Fusarium* fungi, especially *F. graminearum*. These fungi frequently infest multiple field crops, thereby threatening agricultural yields and food safety [5]. ZEN, one of the most detected contaminating mycotoxins usually produced by *Fusarium graminearum*, is classified as an endocrine-disrupting substance due to its capacity to directly bind to and stimulate estrogen receptors (ERs), thus showing an estrogen-like effect [6,7]. FBs are heat-resistant metabolites found in cereal grains (including maize) and crop products, including pelleted feed. The FB1 corresponds to 70% of fumonisins and is produced by *Fusarium verticillioides* [8]. AFs are mainly generated by *Aspergillus flavu* and *Aspergillus parasiticus*, which can contaminate a wide range of food commodities, including nuts (coconut, walnut, pistachio, Brazil nut, and almond), spices (ginger, coriander, turmeric, chilies, and black pepper), oilseeds (cotton, sunflower, soybean, and peanut), cereals (wheat, rice, pearl millet, sorghum, and maize), yam and various milk products [9,10]. The most common metabolic pathway of T-2 is fast deacetylation of the C-4 site, which is then converted into HT-2 toxin and they are all common contaminants in oats, wheat, and corn [11]. These mycotoxins can result in multiple harmful effects, such as genotoxicity, neurotoxicity, nephrotoxicity, hepatotoxicity, gastrointestinal toxicity, reproductive toxicity, and embryotoxicity [2,12,13,14,15]. These detrimental impacts can significantly impair production efficiency and, in severe cases, lead to livestock mortality [16,17,18,19]. Notably, some mycotoxins, such as DON, ZEN, and AFB1, can be detected in various animal-derived products, such as eggs, meat, milk, and processed foods, thereby posing a significant risk to human health through the food chain [20]. A recent comprehensive meta-analysis revealed that feeding DON to broilers can significantly impair their small intestine health and productive performance [16]. Comprehending the precise molecular mechanisms of mycotoxin-induced harmful effects is crucial for advancing potential treatments or detoxification strategies.

Farm animals such as chickens, pigs, ducks, cows, and sheep are mainly exposed to mycotoxins through the consumption of contaminated feed [14]. The gastrointestinal tract usually serves as the first physiological defense against food-borne mycotoxin contaminants [21,22]. Mucosa in the intestinal tissue constitutes a significant functional part of intestinal integrity; an increasing amount of evidence suggests that other elements, such as the microbiota and mucus, are also involved [23]. Multiple studies have shown that exposure to DON, ZEN, FUMs (including FB1, FB2, and FB3), AFB1, and T-2/HT-2 can cause abnormal changes in the function of the intestinal barrier. Additionally, they can also harm the enteric nervous system, disturb the balance of the host’s gut microbiota, and cause the death of intestinal epithelial cells [24,25,26,27,28,29]. The molecular mechanisms of these mycotoxin-induced multiple harmful effects on animal intestinal function are complex and context-dependent [20,23]. They may involve the induction of inflammatory response, cell apoptosis, as well as cell autophagy, the activation of oxidative stress and endoplasmic reticulum stress, the suppression of immune function, and imbalance of gut microbiota in the intestinal tissues [14,29,30,31]. Currently, there is a deficiency in systematic summary and analysis concerning intestinal damage caused by these prominent mycotoxins in farm animals. Thus, in this present review, we mainly summarized the detrimental effects caused by DON, ZEN, FUMs, AFB1, and T-2/HT-2 exposure on the intestinal function in farm animals. Additionally, the potential molecular mechanisms are discussed. Our aim is to put forward new viewpoints regarding intestinal toxicity in farm animals due to mycotoxin exposure, thereby offering a theoretical basis for the development of detoxifying substances.

## 2. An Overview of Mycotoxin-Induced Intestinal Toxicity Effects

Intestinal epithelial cells, together with tight junction proteins and various components, create a physical and biochemical barricade. The intestinal mucosa serves as the first line of defense against these external contaminants, playing a crucial role in maintaining overall health by acting as a barrier to harmful substances. The impact of mycotoxins on the intestinal mucosa is multifaceted, affecting various components of the intestinal barrier, including the mechanical, chemical, immunological, and microbial barriers [32]. The mechanical barrier of the intestinal mucosa is primarily composed of epithelial cells that are tightly joined by proteins such as claudins, which form tight junctions. Mycotoxins can disrupt this barrier by altering the expression and function of these proteins, leading to increased intestinal permeability and compromised barrier integrity. This disruption can facilitate the translocation of pathogens and toxins, potentially leading to systemic infections and inflammation [32]. In addition to the mechanical barrier, mycotoxins can also affect the chemical barrier of the intestinal mucosa, which includes the mucus layer composed of mucins. Mucins are glycoproteins that protect the epithelial surface and maintain mucosal homeostasis. The immunological barrier of the intestinal mucosa is another critical component that can be affected by mycotoxins [21]. These toxins can modulate immune responses by altering the balance of inflammatory cytokines and immune cells, such as lymphocytes, within the intestinal mucosa [21,29,33,34].

It has been shown that DON exposure via oral administration at 2.4 mg/kg body weight for one week significantly decreased the crypt depth and villus height in the intestinal tissues of mice [35]. Pierron et al. showed that DON, not DOM-1 (the diepoxy-metabolite of DON), exposure by gavage at 0.5 nmol per body weight per day for 21 days significantly decreased the villus height in the jejunum of pigs [36]. Recent research has demonstrated that dietary DON exposure at 0.83 mg/kg feed (below the EU-proposed threshold of 0.9 mg/kg feed) can induce villous atrophy and fusion in the intestinal tissues of pigs [37]. Pasternak et al. reported that dietary DON at 3.8 mg/kg body weight for 24 days significantly upregulated the expression of claudin-7, a critical regulator of tight junctions and intestinal homeostasis in weaned piglets [38]. An in vitro proteomic analysis further revealed that 5 μM DON exposure for 24 h substantially modified protein profiles in human Caco-2 cells (a colorectal adenocarcinoma line), particularly affecting cell junction/adhesion molecules. These alterations were partially mediated through suppressed extracellular regulated protein kinase (ERK) and protein kinase A (PKA) pathways [39]. Taken together, this evidence indicates that tight junction proteins may be the potential target of DON exposure. Mechanistically, it has been reported that DON exposure can cause cell apoptosis and inflammation in intestinal tissues through multiple signaling pathways, such as the mitogen-activated protein kinase (MAPK), phosphatidylinositol 3-kinase/Akt (PI3K/Akt), and Janus kinase/signal transducer and activator of transcription (JAK/STAT) pathways [40]. Additionally, DON exposure decreased the expression of β-catenin, and its downstream targets, such as cyclin D1 and Lgr5 proteins in the jejunum tissues of mice, followed by causing intestinal homeostasis dysfunction [41]. This has indicated that DON exposure can damage intestinal mucosal function and the stability of intestinal stem cells.

It is well known that estrogen has various modulatory roles in regulating cellular proliferation and differentiation, modulating intestinal peristalsis, and maintaining normal intestinal physiological functions through the interaction with nuclear estrogen receptor (ER) α/β [6]. ZEN exposure can induce significant toxic effects in the intestines of animals and humans by directly binding to and stimulating ER expression. In a mouse model, exposure to ZEN at 40 mg/kg body weight for 12 days can cause a marked morphological change, which was evident by a reduction in the villus height, an increase in the crypt depth, and a decrease in the ratio of villus length/crypt depth [42]. Very recently, Huangfu et al. found that ZEN exposure at 5 mg/kg body weight per day via the gavage administration for 21 days can significantly disrupt the intestinal barrier, increase intestinal inflammation, disrupt gut microbial diversity, and induce ferroptosis of intestinal epithelial cells in rats [43]. Additionally, ZEN exposure also decreased the expression of claudin-1, ZO-1, and occludin proteins, followed by disturbing the tight-junction structure in the jejunum, and ultimately resulting in the increased permeability of intestinal tissues [42,43]. Lahjouji et al. reported that the exposure to ZEN at 100 μM for 4 h can increase the expression of ERα protein and upregulate the Wnt/β-catenin signaling pathway in the explant culture of jejunum tissue of piglets [6].

It has been demonstrated that exposure to fumonisins can cause various detrimental effects on the intestinal histomorphometry and the expression of intestinal tight junction proteins in farm animals (such as chickens and pigs) [44,45,46,47]. For example, Tomaszewska et al. reported that when hens were orally administered an extract containing FB1 and FB2 at doses of 1, 4, and 10.9 mg/kg body weight for 21 days, it could markedly damage the epithelial integrity of the duodenum and jejunum. Additionally, it significantly reduced the villus height in hens, the ratio of the thickness to the width of duodenal villi, and the ratio of the thickness to the width as well as the depth of the duodenal crypts [44]. Consistently, a recent study reported that oral administration of FB1 (at 5 mg/kg body weight) or hydrolyzed FB1 (at 2.8 mg/kg body weight) for 21 days can both result in compromised integrity, atrophy of intestinal villus, elevated levels of inflammatory factors, and decreased total short-chain fatty acids (SCFAs) in the intestinal tissues in mice [48]. Yu et al. found that FB1 exposure at 10–40 μM for 24 or 48 h can induce dose-dependently cytotoxicity and elevate the ROS accumulation, increase the production of inflammatory factors, disrupt the tight connectivity and permeability, and result in cell apoptosis in IPEC-J2 cells (a porcine intestinal epithelial cell line) [49]. The Nrf2 signal pathway, an endogenous antioxidant stress signaling pathway, was significantly inhibited [50]. Additionally, research has demonstrated that FB1 primarily affects proliferating intestinal cells, leading to lipid peroxidation, inhibition of cell proliferation, and immunomodulatory effects, as evidenced by changes in IL-8 secretion and membrane microviscosity [51].

Around the world, it has been clearly demonstrated that AF contamination poses a significant threat to food safety as well as public health security [3,52]. Among these AFs, AFB1 is the most toxic one [15]. It has been reported that AFB1 exposure can disrupt intestinal microbiota, induce immune responses and oxidative damage, and impair intestinal barrier function and morphology in farm animals [53]. For example, a 21-day exposure to AFB1 at a dose of 0.1 mg/kg body weight can induce ileum tissue damage in ducks. This is manifested by a significantly increased crypt depth and a decreased villus height [54]. Li et al. reported that AFB1 exposure at 0.75 mg/kg body weight once daily for 4 weeks can markedly damage the intestinal barrier and induce an inflammatory response via the induction the dysbiosis of the gut microbiota and inhibition of the AHR signaling pathway in the intestinal tissues of mice [55]. Ding et al. reported that AFB1 exposure can induce pyroptosis and mitochondrial dynamics imbalance via activating NLRP3 and AMPK pathways in IPEC-J2 cells [56].

It was reported that HT-2 toxin exposure at 6.25 nM or T-2 toxin at 3.125 nM for 24 h can significantly decrease the expression of claudin-1, occludin, and ZO-1 mRNAs and proteins in IPEC-J2 cells, indicating damage to the intestinal barrier function [57]. Additionally, the exposure of HT-2 is accompanied by T-2 toxin, and it has been confirmed that the combined exposure of HT-2 and T-2 can induce a significant synergistic toxic effect [57]. Liu et al. reported that T-2 toxin exposure at 1.0, 3.0, and 6.0 mg/kg body weight via the diet for 2 weeks significantly decreased the activities of glutathione peroxidase (GPX), thioredoxin reductase and total antioxidant capacity but increased the concentrations of protein carbonyl and malondialdehyde (MDA) in the duodenum in a dose-dependent manner in the intestinal tissues of chicks. This study also indicated that T-2 toxin-induced intestinal damage is involved in the regulation of nucleotide and glycerophospholipid metabolism, redox homeostasis, inflammation, and apoptosis [58].

The combined exposure to multiple mycotoxins, such as DON, FB1, and ZE, further complicates the toxicological landscape. Studies using metabolomics and lipidomics approaches have shown that these mycotoxins can induce metabolic disorders and damage antioxidant capacity in intestinal cells, with synergistic and antagonistic interactions observed in co-exposure scenarios [59]. It was also reported that the exposure of FB1 at 100 μM or in combination with DON (at 10 μM) can both damage the intestinal morphology (such as the reduction in villus height and the expression of E-cadherin, a junction protein) and the number of goblet cells in jejunal explants from piglets [47,60]. Such findings underscore the importance of considering the cumulative effects of mycotoxin mixtures in risk assessments, as they may exacerbate intestinal toxicity beyond the effects of individual toxins.

In conclusion, these mycotoxin exposures can disrupt the intestinal structure of animals, mainly reflected in the destruction of the intestinal barrier and integrity, as well as its immune function. The intricate molecular mechanisms underlying mycotoxin-induced intestinal toxicity involve the induction of inflammatory reactions and immune dysfunction, the breakdown of the intestinal barrier, the triggering of oxidative stress, and the intestinal microbiota imbalance. These signaling pathways, such as MAPK, Akt/mTOR, TNF, TGF-β, Wnt/β-catenin, PKA, NF-kB, NLRP3, AHR, TLR2, TLR4, IRE1/XBP1, Nrf2, and MLCK pathways, are also implicated. The abnormal expression of micro-RNA also plays a critical role. Continued research in this area is essential for advancing our understanding of mycotoxin-related health risks and for the development of effective interventions to protect human and animal health. In the following section, the detailed molecular mechanisms of mycotoxin exposure-induced intestinal toxicity will be thoroughly discussed.

## 3. Mycotoxin Exposure Triggers Oxidative Stress and Cell Apoptosis

Oxidative stress is commonly regarded as a mechanism in toxic-pathology associated with a variety of adverse environmental agents, like organic compounds, veterinary drugs, and heavy metals [61]. The appearance of oxidative stress is directly related to a rise in the amount of reactive nitrogen species (RNS)/reactive oxygen species (ROS) in cells or tissues [61]. One of the principal sites where ROS/RNS is formed is the gastrointestinal tract [7]. Generally, ROS and RNS encompass a series of oxygen or nitrogen containing free radicals, mainly generated by cell respiration, like singlet oxygen (^1^O_2_), superoxide anion (O_2_^−^•), peroxynitrite (ONOO–), nitrous acid (HNO_2_), hydroxyl radical (OH•), nitrotyrosine, nitric oxide (NO), hydrogen peroxide (H_2_O_2_), and nitrosyl anion (NO−) [62,63]. To keep free radical levels low inside cells, these ROS/RNS can be swiftly cleared or neutralized by the cell’s self-defense antioxidant enzymes (such as GPX, glutathione S transferase [GST], superoxide dismutase [SOD], and catalase [CAT]) or antioxidants (such as reduced glutathione [GSH], selenium, and vitamin C) [3,15]. In normal physiological circumstances, the intracellular states of oxidation and antioxidation remain in an equilibrium. When this balance gets disrupted, there will be a significant rise in intracellular ROS/RNS, which eventually causes oxidative stress damage [2,64].

Similar to other toxic compounds, mycotoxins can also induce oxidative stress damage through conventional pathophysiological mechanisms. Multiple studies have reported that farm animals exposed to mycotoxins might lead to an excess production of ROS/RNS, thereby initiating oxidative stress, finally leading to mitochondrial malfunction and apoptosis, ultimately resulting in intestinal damage [65,66,67]. De Souza et al. reported that DON exposure at 19.3 mg per body weight for seven days markedly promoted the production of ROS and reduced the intracellular GSH levels, subsequently inducing lipid peroxidation in the chickens’ jejunum and ileum tissues [68]. Xu et al. discovered that DON treatment at 0.5 μg/mL for 6–48 h significantly increased the levels of MDA and significantly diminished the activities of CAT and SOD enzymes, then promoted the apoptotic cell death in IPEC-J2 cells (an immortalized intestinal porcine enterocyte cell line) [69]. Consistently, multiple other mycotoxins like HT-2 toxin, AFB1, and ZEN are reported to be able to induce the production of ROS/RNS and interfere with the antioxidant system [70,71]. Sun et al. found that ZEN treatment at 20 μg/mL for 24 h markedly reduced the activities of CAT, GPX, and SOD while markedly increasing intracellular MDA and ROS levels in IPEC-J2 cells [71]. At the same time, it is also reported that ZEN exposure can markedly decrease thioredoxin reductase (TrxR) activities and reduce the levels of intracellular GSH, followed by causing oxidative stress in the intestinal tissues [71]. These results indicated that the overproduction of ROS/RNS or the dysfunction of the antioxidant system-induced oxidative stress plays a critical role in mycotoxin exposure-induced intestinal tissue damage.

When it comes to oxidative stress, the nuclear factor E2-related factor 2 (Nrf2) acts as a housekeeping gene. Additionally, Nrf2 is a crucial transcription factor that directly or indirectly controls the expression of over two thousand genes involved in various biological processes, such as xenobiotic metabolism, phase II detoxification enzyme systems, anti-oxidative stress, anti-inflammatory response, redox-reduction regulation, and drug detoxification [72,73]. A large number of studies have shown that several mycotoxins like T-2, DON, AFB1, and ZEN are able to target and inhibit Nrf2 transcription, thus promoting the downregulation of SOD, GPXs, CAT, and GST at the genetic levels, finally worsening the oxidative stress damage [66,71,74,75,76]. Researchers found that DON exposure at 3 mg/kg body weight for 10 days significantly upregulated the expression of Keap1 and significantly downregulated the expression of Nrf2, then significantly downregulated the expression of heme oxygenase-1 (HO-1) and GPX4 in the jejunum tissues, finally inducing oxidative stress damage [66]. Consistently, Li et al. found that DON exposure at 4 mg/kg via the diet can markedly downregulate the expressions of Nrf2 and its downstream genes glutamate-cysteine ligase modifier subunit (GCLM), glutamate cysteine ligase catalytic subunit (GCLC), HO-1, and NAD (P)H Quinone Dehydrogenase 1 (NQO-1) in the jejunum tissues of piglets [77]. Notably, it was reported that T-2 toxin and AFB1 exposure at the low dose levels can both markedly inhibit the transcription activation of Nrf2 and its downstream antioxidant components in intestinal epithelial cells [74,78]. Nrf2 knockout or knockdown markedly exacerbated AFB1 or T-2 toxin exposure-induced intracellular oxidative stress damage [79,80]. Additionally, it was reported that AFB1 can also selectively impede the Nrf2’s transcriptional activation, then reduce the expression of the downstream detoxification and antioxidant genes, including HO-1, CAT, SOD, GPX, and GST [81,82,83]. Moreover, it was found that T-2 toxin-induced ubiquitination and degradation of Nrf2 is dependent on the activation of activating transcription factor 3 (ATF3) [84]. Unlike T-2 toxin, Xu et al. demonstrated that AFB1-induced the decrease in Nrf2 is dependent on the activation of caveolin-1 protein [85]. This evidence indicate that Nrf2-mediated antioxidant defense plays a vital role in mycotoxin-caused intestinal toxicity. But the underlying mechanisms of different mycotoxins on Nrf2 may be different.

Excessive ROS/RNS production in the animal body can damage intracellular biological macromolecules such as DNA, RNA, proteins, and lipids, ultimately causing programmed cell death [86,87]. Apoptosis, a type of programmed cell death, can be induced in various mycotoxins (such as DON, ZEN, and AFB1)-treated intestinal epithelial cells. It involves the downregulation of B-cell lymphoma-2 (Bcl-2) gene and the upregulation of caspases-3, 8, and 9, Bcl-2-associated x gene (Bax), and tumor suppressor p53 genes and proteins [69,88]. It is widely recognized that a raised Bax/Bcl-2 ratio has the potential to induce mitochondrial dysfunction and initiate the mitochondrial apoptotic pathway through the creation of mitochondrial outer membrane permeabilization (MOMP) [69,88]. The enhanced MOMP can trigger the release of cytochrome C (CytC), then lead to the activation of caspases-9 and -3, and finally induce cell apoptosis [89,90]. Caspases-9 and -3 are usually regarded as the biomarkers of the mitochondrial apoptosis pathway and apoptotic cell death, respectively [89,90]. Xu et al. found that DON treatment at 0.5 μg/mL for six hours markedly increased the Bcl-2 mRNA expression and decreased Bax and caspase-3 mRNA expressions, then induced cell apoptosis in IPEC-J2 cells [69]. In another study, it was found that AFB1 exposure at 30 μg/mL for 24 h markedly increased the mRNA expression of Bax and caspase-3 and decreased the Bcl-2 mRNA expression in IPEC-J2 cells. Similar findings were also observed in AFB1-treated intestinal tissues in mice [91].

Antioxidant supplements can effectively suppress ROS production and oxidative stress resulting from mycotoxin exposure. Subsequently, they can effectively correct mitochondrial dysfunction and the mitochondrial apoptosis pathway caused by mycotoxin exposure. This verifies that oxidative stress is of crucial importance in apoptosis in the intestinal tissues induced by mycotoxin exposure [66,75,92,93]. Additionally, studies have demonstrated that exposure to mycotoxins can trigger cell autophagy to lower ROS levels and relieve the damage caused by oxidative stress. In the end, it safeguards against intestinal damage induced by mycotoxin exposure [94]. It was reported that mycotoxin exposure-induced cell autophagy involves the protein kinase B (Akt) pathway, the p38/MAPK pathway, the IκB kinase (IKK) pathway, and the mammalian target of rapamycin (mTOR) pathway [94,95,96]. For instance, Liu et al. discovered that DON treatment is capable of triggering autophagy through reducing the expression of phosphor-Akt (p-Akt) and p-mTOR while elevating the expression of microtubule-associated protein 1 light chain 3B (LC3-Ⅱ), Beclin-1, and autophagy-related protein 5 (ATG5) mRNAs and proteins in IPEC-J2 cells [96]. Consistently, ATG5 deletion markedly boosts the ROS production, then significantly increases the expression of cleaved-caspase-3 and CytC proteins, finally promoting cell DON exposure-induced cell apoptosis in IPEC-J2 cells. Moreover, it was shown that DON-induced autophagy depends on the activation of IKK and AMPK pathways [95]. In addition, it was also found that the inhibition of ROS production could also significantly block Akt/mTOR-mediated autophagy activation, indicating that DON-induced autophagy is also partly due to the induction of ROS [96].

**Figure 1:**
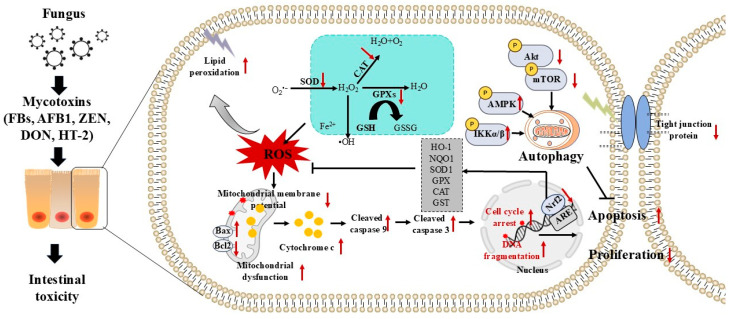
A proposal molecular mechanism model of mycotoxin exposure-induced cell apoptosis and oxidative stress in the intestines of animals. Mycotoxin exposure can promote ROS production via inhibiting SOD, CAT, GPXs activities and reducing GSH levels, then induce lipid peroxidation and oxidative stress. Excessive ROS production can damage mitochondria, cause DNA fragmentation, and cell cycle arrest, finally blocking cell proliferation and inducing cell apoptosis in intestinal epithelial cells. Additionally, mycotoxin exposure can also activate autophagy via the activation of AMPK and IKK pathways and the inhibition of the Akt/mTOR pathway. The inhibition of Nrf2 at the transcriptional level caused by mycotoxin exposure exacerbated oxidative stress damage. Bcl-2, B-cell lymphoma-2; Bax, Bcl-2-associated x gene; AMPK, AMP-activated protein kinase; Akt, protein kinase B; AFB1, aflatoxin B1; CytC, cytochrome C; CAT, catalase; HO-1, heme oxygenase-1; H_2_O_2_, hydrogen peroxide; GST, glutathione S transferase; GSH, glutathione; DON, deoxynivalenol; GPX, glutathione peroxidase; mTOR, mammalian target of rapamycin; IKK, IκB Kinase; SOD: superoxide dismutase; ROS, reactive oxygen species; Nrf2, nuclear factor E2-related factor 2; ZEN, zearalenone.

In short, oxidative stress is extremely important in intestinal damage caused by exposure to mycotoxins. Mycotoxins can lead to mitochondrial malfunction and cell apoptosis by inducing oxidative stress, and this process can be controlled by the Nrf2, Akt/mTOR, IKK, and AMPK pathways (Figure 1). Relieving oxidative stress can efficiently reduce the damage to the intestine caused by mycotoxins, and it has been considered an effective approach to lessen the deleterious effects of mycotoxin exposure on the intestinal tissues of animals.

## 4. Mycotoxin Exposure Triggers the Intestinal Immune Dysfunction and Inflammatory Responses

It is widely recognized that the intestine ranks among the largest immune organs. Moreover, it acts as the chief barrier to ensure animals’ ability to resist natural toxins, which are ingested via the diet or other methods [97]. The immune-regulatory function of the intestine is intricate, and this process involves numerous immune cells and immune-active factors, including T cells producing IL-17 and IL-22, innate lymphoid cells, intraepithelial T cells with innate and cytolytic effector functions, and the production of antimicrobial peptides, among others [98]. Macrophages take in pathogens, display antigens, control the interaction between B lymphocytes and T lymphocytes, and are of central importance in immune regulation [99]. Numerous studies have shown that exposure to mycotoxins can damage intestinal immune function in vitro and animal experiments [100,101,102,103]. It is reported that contact with DON, AFB1, ZEN, FBs, and HT-2 toxin can remarkably decrease cellular immune responses, such as the overall amount of white blood cells, neutrophil phagocytosis activity, macrophage function, and antibody concentration, etc. [104,105,106,107,108]. This is the main reason for the reduction in production capacity and disease resistance among farm animals.

The epoxy group structure of DON can react with the nucleotides present in ribosomal RNA (rRNA). This reaction then disrupts the spatial structure of the ribosome and inhibits the activity of the peptidyl transferase on the 60 S subunit [109]. Ribotoxic stressors can activate the pro-apoptotic as well as pro-inflammatory signaling pathways that are downstream of p38 and JNK signaling pathways [110]. Hu et al. found that DON exposure at 12 mg/kg feed via the diet for 5 weeks significantly increased the expression of p-p38 and p-ERK1/2 proteins and the mRNA expression of several inflammatory factors, including IL-1β, IL-6, IL-8, and TNF-α, and significantly decreased the expression of IL-10 mRNA in the jejunum tissues of mice [111]. Zhang et al. found that DON treatment at 0.5–2 μg/mL can dose-dependently increase expression of p-p38, p-JNK, p-ERK1/2, TNF-α, IL-6, and IL-1α in IPEC-J2 cells. Furthermore, inhibition of p38 can markedly diminish DON exposure-induced upregulation of pro-inflammatory factors, including IL-6, TNF-α, IL-1α, CCL4, CXCL8, CXCL2, IL-12α, CCL20, and IL-15. And the inhibition of ERK 1/2 could notably alleviate the production of DON-induced IL-15 and IL-6, indicating that the activation of p38 and ERK signaling pathways is critical in DON-mediated inflammatory response [112]. Moreover, research has also shown that exposure to the different levels of DON can trigger two different immune effects in vivo as well as in vitro [99]. For instance, DON exposure at the low dose led to intestinal mucosal immune-stimulation, which entailed an increment in the expression of IL-6 and TNF-α mRNA and protein levels, along with a marked increase in the number of T cells and goblet cells. Nevertheless, exposure at the high dose triggered intestinal mucosal immunosuppression, involving an elevation in the mRNA and protein expression of IL-10 and TGF-β genes, and a reduction in the number of T cells and goblet cells in weaned piglets [113]. Moreover, researchers have found that DON exposure at a low concentration can activate the TLR4/NFκB signaling pathway, while DON exposure at high concentration can result in immunosuppression via mitochondrial dysfunction by inhibiting mitophagy [113]. In addition, DON exposure at low doses can also promote inducible nitric oxide synthase (iNOS) ubiquitinoylation and its degradation, resulting in a decrease in NO production in Caco-2 cells, which might potentially account for the enhanced susceptibility of animals to bacterial or viral infection in the intestinal tissues [114]. This is an important factor in explaining DON exposure-induced immune suppression. In another study, it was observed that the humoral and cellular immunity were significantly diminished in pigs fed a DON naturally contaminated diet (i.e., 3.5 mg DON per kg feed) for twenty-eight days in comparison with pigs fed an uncontaminated diet. Additionally, they found that DON exposure significantly upregulated the expression of IL-4 and CXCL10 in the jejunum tissue and the expression of interferon gamma (IFNG) and CXCL10 mRNA in the ileum tissue [115]. Van De et al. discovered that DON treatment at 0.5–5 μg/mL can trigger the activation of NF-κB and the secretion of IL-8 in a dose-dependent manner in Caco-2 cells. Moreover, under pro-inflammatory stimulation, this effect became more intense. This shows that exposure to DON has the potential to lead to or exacerbate intestinal inflammation [116]. Consistently, a recent study showed that DON exposure at the lower dose can trigger the activation of the inflammatory response in IPEC-J2 cells through the activation of the TNF-α/NF-κB/myosin light chain kinase (MLCK) pathway and it may be attributed to the inhibition of the aryl hydrocarbon receptor (AHR) pathway [117].

Jiang et al. found that when broilers are exposed to AFB1 at 0.6 mg/kg feed via the diet, the proportions of CD3+/CD8+, CD3+/CD4+, and CD3+ in the small intestinal T lymphocytes can be decreased. Meanwhile, the mRNA expressions of IL-4, IL-6, IL-10, IL-17, and TNF-α in the duodenal, jejunal, and ileal tissues will also be affected [118]. Furthermore, it was also discovered by them that exposure to AFB1 resulted in a marked decrease in the levels of immunoglobulin A (IgA), immunoglobulin M (IgM), and polymeric immunoglobulin receptor (pIgR) in the serum sample of broilers. Moreover, the mRNA expression of these immunoglobulins, the quantity of mature T cells, and the mRNA expression of IL-6 and IL-2 in the intestinal tissues were also decreased [118]. The evidence presented indicates that the decline in the content of IgA and the expression of inflammatory cytokines could be strongly related to the reduction in the proportion of T-cell subsets brought about by AFB1 exposure. Additionally, AFB1 has been regarded as a non-canonical AHR ligand [55,119]. Zhang et al. discovered that when mice were exposed to AFB1 at 5, 25, and 50 µg/kg body weight daily for thirty days, it could dose-dependently activate the inflammatory response and cause colitis in mice, and the process was partly dependent on the activation of the macrophage AHR/TLR4/STAT3 pathway [119]. In addition, AFB1 treatment can also result in a marked increase in the levels of CD68+ myeloid cells and CD80+ M1 macrophages, and a marked decrease in the levels of CD11b+ cells [119]. Moreover, studies also found that AFB1 exposure can remarkably upregulate the expression of immune-functional proteins, such as indoleamine 2,3-dioxygenase-1 (IDO-1), iNOS, NLRP3, NF-κB, ICAM-1, and COX-2 in the intestinal tissues [119]. Guo et al. discovered that AFB1 exposure at 40 μg/kg body weight via the diet significantly increased the expression of TNF-α, IL-8, IL-6, iNOS, NF-κB, NOD1, and TLR2 mRNAs in the intestinal tissues of broilers [120]. Zhang et al. discovered that a 5-day exposure to AFB1 at a dose of 0.3 mg/kg body weight is capable of triggering an inflammatory reaction in the jejunum and enhancing the expression of TNF-α, IL-6, and IL-1β mRNAs and proteins in the rabbit intestinal tissues [78]. In an another study, it was found that oral AFB1 exposure at 3 mg/kg body weight or AFM1 (a metabolite of AFB1) exposure at 0.3 mg/kg body weight daily for 28 days can both cause a significant increase in the crypt depth, and a decrease in the ratio of villus length/crypt depth in the jejunum tissues of mice [121]. Furthermore, a proteomic analysis revealed that a combination of AFM1 or AFB1 exposure can exacerbate the intestinal barrier dysfunction, and this is positively correlated with several signaling pathways, including the TNF signaling pathway, the microRNAs in cancer, and the IL-17 signaling pathway [121]. These pieces of evidence suggest that immune dysfunction and inflammation in AFB1-treated intestinal tissues are related to multiple signaling pathways, including TLR4, TLR2, NLRP3, AHR, and NF-κB signaling pathways.

Additionally, it has also been reported that ZEN and HT-2 toxin can inhibit intestinal immune function, leading to intestinal damage in farm animals. For example, Wang et al. found that treating with ZEN at 20 mg/kg body weight daily through oral administration for seven days markedly enhanced the IL-1β, IL-10, TNF-α, and IFNG mRNA expressions in the jejunum tissues of mice [122]. Girish et al. showed that dietary Fusarium mycotoxins (including deoxynivalenol at 3.9 μg/g feed, ZEN at 0.67–0.75 μg/g feed, 15-acetyl-DON at 0.34 μg/g feed, and HT-2 toxin at 0.078–0.085 μg/g feed) for 21 days significantly increased the percentage of B-lymphocytes in ileum, and reduced the percentages of CD8(+)-lymphocytes in cecal tonsil in turkey poults [123].

It is also reported that exposure to certain mycotoxins can disrupt intestinal homeostasis and heighten intestinal permeability, which is also referred to as “leaky gut”. Subsequently, this facilitates the translocation of pathogens and lipopolysaccharides (LPS) across the epithelial cell barrier, ultimately leading to gut syndrome [22,124,125]. For example, Awad et al. reported that DON exposure at 5 or 10 mg/kg feed via the diet for five weeks significantly increases the intestinal paracellular permeability in broiler chickens, then promotes the translocation of *Escherichia coli* (*E. coli*) [126]. Verbrugghe et al. found that T-2 toxin exposure can promote the transepithelial passage of *Salmonella* Typhimurium through the intestinal epithelium [127]. Ye et al. reported that AFB1 exposure at low doses can increase serum LPS levels via disrupting the balance of gut microbiota and intestinal barrier structure [128].

Briefly, these mycotoxins can damage the intestinal immune system and induce an inflammatory response, and different mycotoxins have different effects on the expression of inflammation-associated cytokines. They can inhibit the expression of relevant cytokines and genes, reduce the immune response in an inflammatory situation, and increase the susceptibility to diseases. Additionally, mycotoxin can also promote the translocation of intestinal pathogens and LPS through the epithelial cell barrier, leading to induce gut syndrome. Mycotoxin-regulated immune modulation and inflammatory response in the intestinal tissues may be implicated in multiple signaling pathways, including TGF-β, NF-kB, NLRP3, AHR, TLR2, TLR4, MAPK, IRE1/XBP1, and MLCK pathways. These findings provide important intervention targets for the development of intervention strategies to alleviate intestinal damage due to mycotoxin exposure.

## 5. Mycotoxin Exposure Causes Disruption of Intestinal Microbiota

The intestine is a vital digestive organ, and its stable, functional mucosal barrier serves as the biological interface separating the body’s internal environment from food antigens and microorganisms. The gut microbiota, the largest microecological system, plays a crucial role in maintaining a stable internal and external environment. Disruption of the intestinal microbiota is primarily characterized by an imbalance in the growth of symbiotic and pathogenic bacteria and is closely associated with disease progression [129]. Generally, the composition and function of intestinal microbiota can be disturbed by various factors, including genetic factors, antibiotics, diet, and environmental chemicals [130]. Intestinal microbiota can modulate the endocrine functions and energy absorption; therefore, it plays a key role in host metabolism [131,132,133,134,135]. It has been reported that mycotoxins can be biologically inactivated or degraded by the intestinal microbiota [136,137]. Nevertheless, excessive exposure to mycotoxins can disrupt the homeostasis of the intestinal microbiota and impede the absorption of intestinal nutrients.

DON exposure at the low dose (i.e., at 10 µg/kg body weight daily) via the diet for 280 days can markedly induce the changes in the microbial composition in the mouse intestine; a remarkable rise was observed in the relative abundances of *Cyanobacteria*, *Tenericutes*, *Verrucomicrobia*, TM7, *Proteobacteria*, *Deferribacteres*, and a substantial decline was observed in the relative abundances of *Actinobacteria* and *Bacteroidetes* [138]. Furthermore, exposure to DON can significantly raise the quantity of *Mucispirillum* as well. These bacteria are capable of degrading mucin and may play a role in the already-known impacts of DON on the intestinal barrier [138]. It has also been demonstrated that *Lactobacillus* and *Bacteroides* can also eliminate DON and ZEN in the intestinal tissue [139,140]. Therefore, leveraging the antibacterial properties of beneficial bacteria can effectively relieve the harm of mycotoxins to intestinal functions.

Piotrowska et al. found that ZEN treatment separately or together with DON can both cause a marked effect on mesophilic aerobic bacteria, and significantly reduces the relative enrichment of *E. coli* and *Clostridium perfringens* (*C. perfringens*) in the intestinal tissues of porcine [141]. The enrichment of bacteria containing lipopolysaccharide (LPS) may be an important cause of systemic inflammation and immune response. In addition, a recent study showed that T-2 toxin treatment can promote *E. coli* to develop stable resistance to multiple clinical antibiotics, such as cephalosporins, carbapenems, tigecycline, and colistin [142]. This indicates that mycotoxin exposure not only increases the susceptibility of the intestine to pathogenic bacteria but may also enhance bacterial resistance, thereby reducing the effectiveness of clinical antibiotic treatment.

It is widely acknowledged that intestinal microbiota dysbiosis can affect gut health through the metabolites such as SCFAs (i.e., acetate, propionate, and butyrate), LPS, bile acid, and small peptides [143]. Notably, alterations in the levels of SCFAs and LPS play a vital role in regulating intestinal NLRP3 signaling pathways [144]. In the above-mentioned, the intestinal toxicity caused by NLRP3 activation upon exposure to T-2 and ZEN has been described. Therefore, we believe that exposure to these mycotoxins may lead to a reduction in SCFAs and an increase in LPS by inhibiting the growth of probiotics and promoting the growth of pathogenic bacteria, which triggers excessive NLRP3 activation and results in intestinal inflammation. It has also been reported that DON exposure could cause the disruption of intestinal microbiota, and then lead to excessive secretion of peptide YY (PYY) and 5-HT, which are two key neurotransmitters in controlling emetic [145]. It is known that this abnormal PYY and 5-HT secretions can directly or indirectly inhibit or activate multiple signaling pathways, such as NF-κB, MAPK, TLR pathways, and cell apoptosis and autophagy in intestinal epithelial cells [146,147]. This indicated that the specific intestinal toxicity of DON may be closely related to its effect on intestinal nerve cells. Additionally, it reported that DON exposure can inhibit intestinal bile acid reabsorption and lead to bile acid malabsorption in the intestine [148]. Bile acids can stimulate mitochondria to produce excessive ROS by disrupting the respiratory complexes and electron chain transfer in mitochondria [149]. This evidence indicated that DON can induce intestinal oxidative damage via the induction of the accumulation of bile acids in the intestinal tissues.

**Figure 2:**
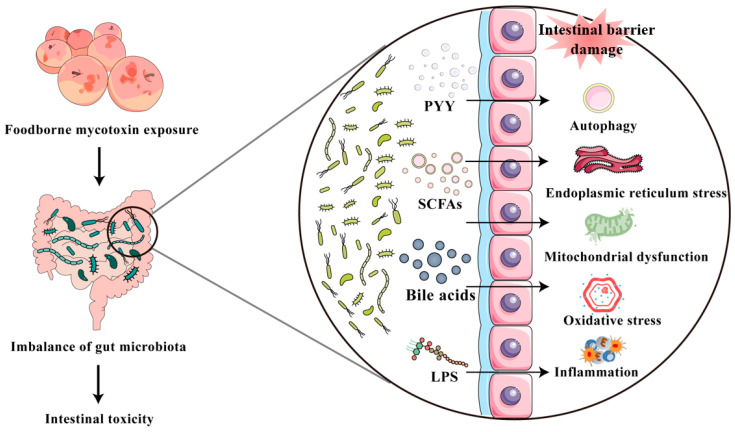
Mycotoxin exposure can disrupt the intestinal micro-ecological balance, subsequently resulting in a reduction in short-chain fatty acid (SCFA) production, an increase in LPS, an excessive secretion of peptide YY (PYY), and an accumulation of bile acids, initiating a cascade to induce endoplasmic reticulum stress, mitochondrial dysfunction, oxidative stress, and inflammatory response, finally resulting in intestinal barrier injury.

In brief, mycotoxin exposure can disrupt the intestinal micro-ecological balance, subsequently result in the enrichment of harmful metabolites or reduction in beneficial metabolites, leading to disturbance of the intestinal barrier and permeability and ultimately result in inflammatory responses, oxidative stress, immune dysfunction, and other adverse health effects (Figure 2). Moreover, the mechanisms by which different mycotoxins disrupt the homeostasis of intestinal microbiota suggest special relationships between mycotoxins and intestinal microbiota. This may be due to different mycotoxins having specific antibacterial properties and the interactions between different mycotoxin entities and the intestinal microbiota in certain specific environments. Currently, some studies have confirmed that certain intestinal microbiota, such as commercially available lactic acid bacteria or probiotics isolated from animal intestines, can effectively ameliorate intestinal microbiota dysbiosis induced by mycotoxin exposure and other health-damaging effects [150,151,152,153]. In addition, future research exploring the effects of mycotoxins on a specific bacterial genus to understand its specific mechanism of action is required.

## 6. Role of MiRNAs in Mycotoxin-Induced Intestine Toxicity

MicroRNAs (miRNAs) belong to a type of endogenous non-coding small molecular substances, and their lengths are approximately 21–28 nt. They can regulate gene expression post-transcriptionally, influencing various biological processes, including those related to toxicology. [154]. miRNAs are regulators whose up- or downregulation acts on the development of pathological dysregulation. MiRNAs also participate in regulating multiple signaling pathways to govern various mycotoxin exposure-induced toxicity and tissue damage [155].

The role of miRNAs in mycotoxin-induced intestinal toxicity is an emerging area of research that underscores the complex interplay between genetic regulation and toxicological responses. A great number of studies have shown that miRNAs can act as nodes within signaling networks and play a crucial role in maintaining homeostasis and controlling various types of cell death, including pyroptosis, ferroptosis, and apoptosis, as well as chronic diseases like fibrosis, metastasis, neurodegenerative diseases, cardiovascular diseases, and cancer [156,157]. Variations in microRNA expression have been detected following exposure to different mycotoxins, primarily resulting from altered regulatory mechanisms post-toxin contact and interspecies disparities in miRNA functions [158]. It was reported that ZEN exposure can upregulate the expression of miR-452-3p, miR-424-5p, and miR-1 in piglets [159]. Chuturgoon et al. reported that FB1 exposure can significantly upregulate the expression of CYP1B1 via the target inhibition of miR-27b [160]. Rieswijk et al. demonstrated that AFB1 exposure can upregulate the expression of miR-301b-3p to regulate cell cycle arrest and DNA damage [161]. Additionally, it also reported that AFB1 exposure can upregulate the expression of miR-24, miR-33a, miR-34a, miR-34a-5p, and downregulate the expression of miR-138-1 and miR-122, following to regulate cell proliferation, DNA damage, or cancer generation [158,162,163,164]. A systematic review on the impact of several different mycotoxins (e.g., DON, ZEN, and AFB1) on miRNAs and the underlying toxicology mechanisms has been performed by Chen et al. [155] and Rong et al. [158].

Collectively, these studies provide a comprehensive view of the multifaceted roles of miRNAs in mycotoxin-induced intestinal toxicity. This highlights the potential of miRNAs as diagnostic biomarkers and therapeutic targets, offering new avenues for research and intervention in the management of mycotoxin-related health risks. By integrating insights from various studies, it becomes evident that miRNAs play a crucial role in mediating the toxicological and protective responses to mycotoxin exposure, thereby shaping the future of food safety and toxicology research.

## 7. Summary and Prospect

Mycotoxin exposure can damage the integrity of the intestinal morphological structure, cause intestinal dysfunction, and finally lead to a decrease in the production performance or products of farm animals. Various mycotoxins like DON, ZEN, FBs, AFB1, and HT-2 toxin may have different levels of impact (both molecular and tissue level), and their mechanisms of action are complex. Studies indicate that the possible mechanisms behind mycotoxin exposure-induced abnormal intestinal function involve the breakdown of the intestinal barrier, the triggering of an inflammatory response and immune malfunction, the activation of oxidative stress, and the imbalance of the intestinal microbiota. Multiple signaling pathways, including TGF-β, PKA, MAPK, TNF, NF-κB, NLRP3, AHR, TLR2, TLR4, IRE1/XBP1, Nrf2, Akt/mTOR, Wnt/β-catenin, and MLCK pathways, are also implicated. These findings provide important theoretical foundations for intervention strategies against these mycotoxin-induced intestinal toxicities. The targeting of the reduction in mycotoxin exposure from agricultural practice to food/and feed additives is also required.

The general body of literature highlights several aspects for future research: (i) It is crucial to investigate the potential toxic effects and the exact molecular correlations between exposure to multiple mycotoxins and other food contaminant-caused intestinal toxicity in animals and humans. (ii) Importantly, the abnormal expression of miRNA can disturb various signal transduction and seems to play a critical role in mycotoxin-caused intestinal dysregulation. Currently, the precise molecular mechanisms are incompletely understood, and more investigations are required. (iii) As demonstrated by large-scale investigations, both humans and animals are simultaneously exposed to several mycotoxins, such as AFB1, DON, ZEN, HT-2/T-2, or FUNs. The exposure to several mycotoxin combinations may lead to synergistic, cumulative, or antagonistic effects. Certain mycotoxins, such as DON and AFB1 combined, might produce significant synergistic toxic effects. Consequently, molecular mechanism studies focused on single mycotoxins might not be suitable for the joint exposure of multiple mycotoxins. (iii) Previous studies have mainly centered on the influence of these mycotoxins on gut microbiota but might have overlooked the effect of mycotoxins on the formation and dissemination of bacterial resistance, which could directly affect clinical drug treatment and even pose a threat to public health safety. More attention is needed in future research. (iv) There are still many unknown areas in the research around this ‘mycotoxin–gut microbiota–metabolite-signaling pathways’ metabolic axis, and in-depth research is urgently needed.

## Data Availability

All data are included in this manuscript.

## Abbreviations

The following abbreviations are used in this manuscript:

Akt, protein kinase B; AFs, aflatoxins; AHR, aryl hydrocarbon receptor; ATG5, autophagy-related protein 5; AMPK: AMP-activated protein kinase; Bax, Bcl-2-associated x gene; Bcl-2, B-cell lymphoma-2; CytC, cytochrome C; CAT, catalase; Caco-2, human epithelial colorectal adenocarcinoma; DAS, 4,15-diacetoxyscirpenol; DON, deoxynivalenol; ER, estrogen receptors; eNOS, endothelial nitric oxide synthase; ERK1/2, extracellular regulated protein kinase 1/2; FUMs, fumonisins; GST, glutathione S-transferase; GSH, glutathione; GR, glutathione reductase; GPX, glutathione peroxidase; H_2_O_2_, hydrogen peroxide; HNO2, nitrous acid; HO-1, heme oxygenase-1; IgA, immunoglobulin A; IgM, immunoglobulin M; IKK, IκB Kinase; iNOS, inducible nitric oxide synthase; LPS, lipopolysaccharide; LC3-II, microtubule-associated protein 1 light chain 3B; mTOR, mammalian target of rapamycin; MDA, malondialdehyde; MOMP, mitochondrial outer membrane permeabilization; MLCK, myosin light chain kinase; MAPK, mitogen-activated protein kinase; NEO, neosolaniol; NIV, nivalenol; NO, nitric oxide; NO−, nitrosyl anion; Nrf2, nuclear factor E2-related factor 2; ONOO–, peroxynitrite; O_2_^−^•, superoxide anion; OTA, ochratoxins; OH•, hydroxyl radical; ^1^O_2_, singlet oxygen; pIgR, polymeric immunoglobulin receptor; PYY, peptide YY; PKA, protein kinase A; rRNA, ribosomal RNA; RNS, reactive nitrogen species; ROS, reactive oxygen species; TCN, trichothecin; TrxR, thioredoxin reductase; TNF, tumor necrosis factor; VIP-LI, vasoactive intestinal polypeptide-like immunoreactive; ZEN, zearalenone; ZO-1, zonula occludens-1; XBP1: X-box-binding protein 1; SCFAs, short-chain fatty acids; SOD, superoxide dismutase.
